# Development of a longevity prediction model for cut roses using hyperspectral imaging and a convolutional neural network

**DOI:** 10.3389/fpls.2023.1296473

**Published:** 2024-01-10

**Authors:** Yong-Tae Kim, Suong Tuyet Thi Ha, Byung-Chun In

**Affiliations:** Department of Smart Horticultural Science, Andong National University, Andong, Republic of Korea

**Keywords:** cut roses, deep learning, gray mold disease, hyperspectral imaging, prediction, vase life

## Abstract

**Introduction:**

Hyperspectral imaging (HSI) and deep learning techniques have been widely applied to predict postharvest quality and shelf life in multiple horticultural crops such as vegetables, mushrooms, and fruits; however, few studies show the application of these techniques to evaluate the quality issues of cut flowers. Therefore, in this study, we developed a non-contact and rapid detection technique for the emergence of gray mold disease (GMD) and the potential longevity of cut roses using deep learning techniques based on HSI data.

**Methods:**

Cut flowers of two rose cultivars (‘All For Love’ and ‘White Beauty’) underwent either dry transport (thus impaired cut flower hydration), ethylene exposure, or *Botrytis cinerea* inoculation, in order to identify the characteristic light wavelengths that are closely correlated with plant physiological states based on HSI. The flower bud of cut roses was selected for HSI measurement and the development of a vase life prediction model utilizing YOLOv5.

**Results and discussion:**

The HSI results revealed that spectral reflectance between 470 to 680 nm was strongly correlated with gray mold disease (GMD), whereas those between 700 to 900 nm were strongly correlated with flower wilting or vase life. To develop a YOLOv5 prediction model that can be used to anticipate flower longevity, the vase life of cut roses was classed into two categories as over 5 d (+5D) and under 5 d (-5D), based on scoring a grading standard on the flower quality. A total of 3000 images from HSI were forwarded to the YOLOv5 model for training and prediction of GMD and vase life of cut flowers. Validation of the prediction model using independent data confirmed its high predictive accuracy in evaluating the vase life of both ‘All For Love’ (r^2^ = 0.86) and ‘White Beauty’ (r^2^ = 0.83) cut flowers. The YOLOv5 model also accurately detected and classified GMD in the cut rose flowers based on the image data. Our results demonstrate that the combination of HSI and deep learning is a reliable method for detecting early GMD infection and evaluating the longevity of cut roses.

## 1 Introduction

Recently consumer interest and use of floricultural products have been growing, especially through online markets, resulting from an increase in flower sale for home use in the COVID-19 era (Bulgari et al., 2021; Gabellini and Scaramuzzi, 2022). As a large portion of floricultural plants is utilized as cut flowers, long postharvest longevity is the primary quality by which flower sales can be promoted (Vehniwal and Abbey, 2019). Although cut flower longevity cannot be readily assessed, estimates of shorter vase life commonly reduce the value of cut flowers that are shipped to international markets. The vase life of cut flowers is determined by morphological and physiological attributes, which are shaped by the interaction of preharvest conditions and genetic traits (Fanourakis et al., 2013; In and Lim, 2018). Although rose is not an ethylene-sensitive species, in some cultivars adverse effects of ethylene exposure have been reported (Macnish et al., 2010; In et al., 2017). Ethylene is a plant hormone that regulates various physiological processes, including fruit ripening and flower senescence (Wang et al., 2002). Ethylene is also produced as a product of certain agricultural commodities and industrial activities. Cut rose flowers can be exposed to increased ethylene concentrations in various situations such as storage or transport with ethylene-producing agricultural commodities (fruits or flowers that naturally produce ethylene), storage in or near industrial areas where there is high emission of the ethylene-producing substances, and the improper ventilation of the storage or transport facilities (Cape, 2003; Chang and Bleecker, 2004; Martínez-Romero et al., 2009). Dry transport is the main method employed commercially for trade because of a reduction in space (thus cheaper) and in flower bud opening (thus maturity stage is little affected) (Macnish et al., 2009). However, cut flower hydration during dry transport is reduced owing to transpiration losses, which are not compensated. In addition, some environments such as high humidity and wet conditions are characterized by increased *B. cinerea* spore density (Williamson et al., 2007; Friedman et al., 2010). This increased spore density is not apparent at harvest, but later on, problems appear.

Therefore, the vase life of cut roses commonly ends during the early stages of flowers’ development, and reliably predicting their lifespan has not been possible. Consequently, consumers are dissatisfied and flower utilization is reduced (Reid et al., 1996; Vehniwal and Abbey, 2019). Thus, the development of longevity prediction techniques is a high priority to assure the ornamental period of cut flowers for the customers, as this can be incorporated into the existing system for quality grading of cut flowers. Moreover, the vase life prediction system can improve efficiency in flower supply chains as well as provide consumers with relevant flower products according to their use. For instance, different batches of cut flowers can be sorted based on the vase life potential in the packaging house. The flower batches with short vase life are priced lower and traded shortly, whereas the batches with long vase life are priced higher in the markets and can be stored for longer time before the distribution. Furthermore, the vase life prediction model in cut flowers offers benefits ranging from quality assurance and supply chain optimization to environmental sustainability and economic efficiency. It aligns with the boarder goals of the floral industry, aiming to deliver high quality products while minimizing waste and environmental impact.

Few attempts had so far been made to devise effective methods to predict and guarantee postharvest longevity of cut flowers. Staby and Cunningham (1980) reported a method to estimate the vase life of cut carnation based on the ethylene level using gas chromatography. However, vase life prediction using this method is not suitable in ethylene-insensitive flowers and might be less accurate in the early stage of postharvest. Tromp et al. (2012) developed a method to predict the remaining vase life of cut roses using the degree-days model during storage and transportation at a constant. However, this method may be of limited use if the biological variance is high or the temperature of storage and transportation is outside the optimum range (2-6 °C).

We developed previously artificial neural network models to predict and assure the vase life of three rose cultivars based on thermal image analysis. Although the prediction accuracy of the models was quite high, the application of this method was limited because the cut roses used for the prediction model did not undergo various postharvest conditions that influence the vase life of cut flowers, such as dry transport, exposure to ethylene, or high density of mold spore during storage and transport (In et al., 2009; In et al., 2016a). Thus, to enhance the model performance for practical application in the vase life guarantee, it is further necessary to detect plant status rapidly and to use extensive data processing for complex data, such as artificial intelligence or machine learning.

Recently, a non-destructive method such as hyperspectral imaging (HSI) has been widely used to evaluate various factors related to plant physiology and stress conditions in multiple horticultural crops (Behmann et al., 2014; Liu et al., 2015; Lowe et al., 2017; Veys et al., 2019; Ramamoorthy et al., 2022; Wieme et al., 2022). HSI uses a hyperspectral camera to capture images of plants in a wide range of light wavelengths (Lowe et al., 2017; Lay et al., 2023). By analyzing the reflectance of horticultural products in different wavelengths, HSI can extract detailed information about the morphological and physiological properties of plants, including disease infection, nutritional deficiencies, ripeness, and defects of fruits and vegetables, etc (Liu et al., 2015; Wieme et al., 2022). The development of spectral imaging techniques has required suitable regression models to analyze spectral data. Machine learning techniques based on algorithms have been applied to construct classification and regression models for HSI to predict and evaluate the quality of vegetables and fruits (Zhang et al., 2016; Rahman et al., 2017; Ji et al., 2019). However, the machine learning algorithms only performed a screening process on the spectral bands (Zhang et al., 2016). In recent years, deep learning, a subset of machine learning, has been widely used in agriculture, industry, and medics because it can learn features automatically from a large dataset of images (Guo et al., 2016; Tian et al., 2020). This technique was used in building hyperspectral imaging correction models for prediction and classification. Convolutional neural networks (CNNs), a type of deep learning algorithm, can rapidly and accurately classify the quality of agricultural products and identify potential factors affecting their appearance or shelf life without being influenced by personal biases or subjective opinions (LeCun et al., 2015; Kamilaris and Prenafeta-Boldú, 2018; Cravero et al., 2022). In the last decade, CNNs have been increasing employed in plant phenotyping community. They have been very effective in modeling complicated concepts, owing to their ability of distinguishing patterns and extracting regularities from data (Nasiri et al., 2021; Taheri-Garavand et al., 2021). You Only Look Once version 5 (YOLOv5), a type of CNN, is a state-of-the-art deep learning algorithm that was used to classify agricultural products with high accuracy even when source images are poor quality or contain multiple features (Yao et al., 2021; Ahmad et al., 2022). To classify agricultural products by using YOLOv5, the algorithm must first be trained on a large dataset of labeled images (Redmon et al., 2016; Yao et al., 2021). YOLOv5 can also perform real-time classification, which is important for rapidly classifying large quantities of horticultural products (Zhang et al., 2021; Li et al., 2022). HSI and deep learning techniques have been widely applied to predict postharvest quality and shelf life in multiple horticultural crops such as vegetables, fruits, and mushrooms (Taghizadeh et al., 2011; Mo et al., 2015; Susič et al., 2018; Sun J et al., 2021; Wieme et al., 2022; Xiang et al., 2022); however, there are few studies showed the application of these techniques to evaluate the quality issues of cut flowers (Stead et al., 2018; Sun X et al., 2021). Therefore, this study aimed to develop a rapid and effective method to predict the longevity of cut roses based on HSI and deep learning algorithms. To identify light wavelengths that are closely correlated with plant physiological states (GMD and petal wilting) using HSI, cut flowers underwent either water stress, ethylene exposure, or *B. cinerea* inoculation before storage. YOLOv5 was adopted for processing the extensive image data by HSI in order to develop vase life prediction models for cut flowers. In the present study, the flower bud of cut roses was chosen for HSI measurement and the development of the vase life prediction model. This selection allows for imaging from the top of entire batches of cut flowers. Furthermore, the results obtained in this study are not confined solely to hydration status; they also contribute to the vase life prediction for cut rose flowers.

## 2 Materials and methods

### 2.1 Plant materials

Cut roses ‘All For Love’ and ‘White Beauty’(*Rosa hybrida* L.) were cultivated and harvested in a commercial greenhouse in Guksong, Jeollanam-do, South Korea. Rose plants were dripirrigated with a liquid nutrient solution containing NH_4_NO_3_ (44.93 _g_ L^-1^), Ca(NO_3_)_2_ 4H_2_O (17.47 g L^-1^), KNO_3_ (1.63 _g_ L^-1^), KH_2_PO_4_ (12.04 _g_ L^-1^), MgSO_4_ 7H_2_O (27.04 g L^-1^), and a small volume of other trace substances. The symptomless rose flowers were collected and harvested at the commercial stage (outer petals bent out) (Harkema et al., 2013). After harvest, cut flowers were either wet transported (WT) in tap water or dry transported (DT) without the water to the laboratory within 4 h. At the laboratory, all cut roses were placed in a controlled environment room at 23 ± 1 °C and at a relative humidity (RH) of 50 ± 2% for HSI analysis. After the HSI, the cut flowers were exposed to ethylene or inoculated with *B. cinerea* and followed by storage at 10 ± 1 °C and RH of 50 ± 5% under dark conditions for 3 d for transport treatments (In et al., 2016b).

### 2.2 Ethylene exposure

Cut flowers in WT were held in distilled water and those in DT were placed in buckets without water and enclosed in the treatment chamber (462 L) at 23 ± 1 °C under dark conditions. Distilled water was used, though less common from practical stand point, since the tap water composition largely depends on the season, and the location (Amadi-Majd et al., 2021). Ethylene (10%) was injected into the chamber to achieve a final concentration of 2 µL L^-1^. Three beakers containing 200 mL of 1M NaOH were placed in the treatment chamber to neutralize CO_2_ released by the flower respiration during the ethylene treatment. After every 12 h of ethylene exposure, the treatment chamber was opened for 2–3 h for HSI and then closed and re-injected with 2 µL L^-1^ ethylene. Three days after the transport treatments, cut flowers were taken out from the chamber for vase life evaluation and HSI.

### 2.3 *B. cinerea* inoculation in cut roses

*B. cinerea* (KACC40573) was isolated from infected rose flowers in the Korean Agricultural Culture Collection (KACC), National Institute of Agricultural Sciences. For a pure culture, *B. cinerea* conidia were grown in potato dextrose agar (PDA, Difo Laboratories, Detroit, MI, USA) at 25 ± 1 °C for 14 days. *B. cinerea* conidial suspension was obtained by dropping 10 mL of distilled water into a culture petri dish and then gently sweeping the fungal colony surface with a sterile loop. The conidial clumps were removed from the obtained suspension by gently filtering with sterile gauze. Afterward, the concentration of conidia suspension was adjusted to 10^5^ conidia mL^-1^ with sterile water for the experiment.

WT and DT flowers were inoculated by spraying with 30 mL of the conidial suspension (10^5^ conidia mL^-1^). Non-inoculated cut roses were sprayed with sterile water (30 mL). After inoculation of *B. cinerea*, the rose flowers were then placed in the storage chamber (at temperature 10 ± 1 °C and RH of 50 ± 5%) under dark conditions for 3 d to simulate export conditions. After the transport treatments, cut flowers were set up for vase life and disease progression evaluation and HSI.

### 2.4 Evaluation of vase life and gray mold disease

After three days of the export simulation, twenty-five cut roses in each treatment were trimmed to a length of 45 cm with three upper leaves. Each cut flower was placed into a glass jar containing distilled water (450 mL) and maintained at the temperature (23 ± 1 °C), RH of 50 ± 2%, and light intensity at 20 µmol m^-2^ s^-1^ (a photoperiod of 12 h) supplied by fluorescence tubes for GMD progression and vase life assessment.

Changes in the postharvest quality of cut roses were determined by measuring relative fresh weight and water uptake daily at 10:30 am. Water balance (WB) of cut flowers was calculated from changes in fresh weight, water uptake, and daily transpiration. The vase life of cut roses was evaluated daily by the assessment criteria for *Rosa* (VBN, 2014). Cut roses were considered to have reached the end of their postharvest life when flowers showed at least one or more of the following senescence symptoms: pedicel bending (neck angle greater than 45°), petal drying (≥ 50% of petals show dryness); wilting of petal and leaf (≥ 50% of petals or leaves loss their turgor), petal abscission (a drop of three or more petals), leaf abscission and yellowing (≥ 50% leaf drop and yellowing), bluing (≥ 50% blue petals) (Fanourakis et al., 2015; Fanourakis et al., 2016). In addition, the vase life of cut roses was considered to end when cut flowers showed severe GMD symptoms in the petals. The progression of GMD by *B. cinerea* was evaluated based on the disease index as described in the previous study (Ha et al., 2022).

### 2.5 Fungal biomass and gene expression analysis

Fungal genomic DNA (gDNA) was extracted from the gray mold mycelia collected from infected petals by using i-genomic BYF DNA Extraction Mini Kit (INTRON Biotechnology Inc., Gyeonggi-do, South Korea). Total RNA was isolated from 200 mg of rose petals by using the GeneJET plant RNA purification Mini Kit (Thermo Fisher Scientific Baltics, Vilnius, Lithuania). cDNA was synthesized from 1 µg of total RNA using XENO-cDNA Synthesis Kit (CELL TO BIO, Gyeonggi-do, South Korea) and performed in a Bio-Rad PTC-100 Programmable Thermal Controller (MJ Research Inc., Hercules, CA, USA) as per the instruction manual. Then, fungal biomass (evaluated by *Bc3* from gDNA) and the transcript levels of the ethylene biosynthesis gene (*RhACO1*), aquaporin-related gene (*RhTIP1*), and senescence-induced gene (*RhSIG*) in petals of cut roses were analyzed using the BIO-RAD CFX Connect Real-Time System (Life Science, Hercules, CA, USA). *B. cinerea* actin A (*BcactA*) and *Rosa hybrida* actin 1 (*RhACT1*) genes were used as an internal control. The primer sequences used for quantitative real-time PCR (qRT-PCR) are listed in **Table 1**. The qRT-PCR reaction setting and conditions for gene expression analyses have been indicated previously (Ha et al., 2022).

**Table 1:** List of genes and primers used for qRT-PCR analysis in this study.

Gene (accession number/reference)	Forward primer	Reverse primer
*Bc3* (Suarez et al., 2005)	5’-GCTGTAATTTCAATGTGCAGAATCC-3’	5’-GGAGCAACAATTAATCGCATTTC-3’
*BcactA* (Chagué et al., 2006)	5′-CCCAATCAACCCAAAGCTCAACAG-3′	5′-CCACCGCTCTCAAGACCCAAGA-3′
*RhACO1* (AF441282.1)	5′CGTTCTACAACCCAGGCAAT-3′	5′-TTGAGGCCTGCATAGAGCTT-3′
*RhTIP1* (KF985188.1)	5’-TCTCTCCTACGTGGCATCCT-3’	5’-GACCACCTCTGCTTTTGCTC-3’
*RhSIG* (S80863.1)	5’-CCGACACAAGAACCTTGGAT-3’	5’-TCTTCCGTGTACACCACCAA-3’
*RhACT1* (KC514918.1)	5′-GTTCCCAGGAATCGCTGATA-3′	5′-ATCCTCCGATCCAAACACTG-3′

### 2.6 Hyperspectral image acquisition

The visible/near-infrared (VIS-NIR) hyperspectral camera system was set with an IMEC SNAPSCAN camera (3650x2048 pixel) (IMEC, Leuven, Belgium, www.imec-int.com) with 150 spectral bands and a spectral range of 470–900 nm. This system was connected to a computer (Intel (R) Core (TM) I7-1165G7 CPU @ 2.8 GHz). Images of cut roses were acquired using the HSI in reflection mode and were constructed under a controlled environment room (23 ± 1 °C and RH of 50 ± 2%). The VNIR light source was provided by 4 halogen Osram lamps with 20W HT spot and color temperature of 2800 K (OSRAM, Munich, Germany). The halogen lamps provide 350-2500 nm light with a power of 20 W. The distance between the cut rose flowers and the lens was set to 50 cm, and the angle between the lamps and camera was set at 45° to provide enough light to the imaging area for image acquisition. The exposure time of the hyperspectral camera shooting was set to 2 milliseconds. The halogen lamps were run for 15 min to reach a stable state temperature and then a 95% reflection standard was calibrated before conducting reflection measurements of the cut roses. Data acquisition and extraction were performed using the IMEC HSI Snapscan software version 1.8.1.1 (IMEC, Leuven, Belgium).

### 2.7 Image processing model

**Figure 1:**
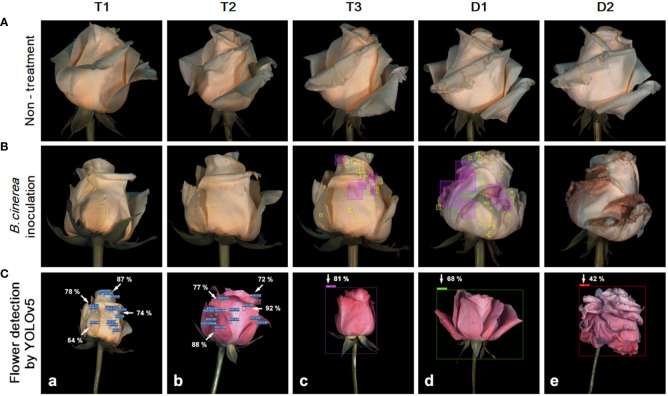
Development of flower opening and gray mold disease (GMD) in ‘White Beauty’ cut roses during transport and vase life **(A, B)**. The cut flowers were untreated (none treatment) or sprayed with *B. cinerea* conidia suspension (inoculation) and the images of flowers were taken on days 1-3 of transport (T1-T3) and days 1-2 (D1-D2) of vase period. The green and pink boxes indicate the annotation of the GMD emergence spots by bounding boxes for deep learning analysis **(B)**. Detection of GMD and petal wilting in ‘White Beauty’ **(a)** and ‘All For Love’ **(b–e)** by YOLOv5 **(C)**. The arrows and numbers in the flower images indicate the GMD spots and the probability (%) of GMD calculated by YOLOv5 **(a, b)**. The bounding boxes in purple, green, and red generated by annotation tool MAKE SENSE indicate petal wilting and opening levels of the flowers at T0, T3, and D1. The percentage numbers in the images indicate the probability of the specific wilting and opening stages, as calculated by YOLOv5 **(c–e)**.


A dataset of images of cut roses was used to process disease detection and vase life prediction by using deep learning system YOLOv5 version 6.2 (GitHub, San Francisco, USA). The dataset consisted of 3000 images collected from the hyperspectral system, with 1500 disease-infected cut roses and 1500 non-disease-infected cut roses. The images were resized to 640x640 pixels and the disease-infected areas in the images were annotated with bounding boxes using MAKE SENSE (**Figures 1A–C**). The annotation process was done by a trained 1 annotator is familiar with disease-infected cut roses to ensure consistency and accuracy. The YOLOv5 architecture implemented in Python using the PyTorch library was used for object detection. The YOLOv5x model was implemented using the GitHub library and was trained on a computer with a CUDA-enabled GeForce RTX 3080 graphics card for 50 epochs. To evaluate the performance of gray mold disease detection in cut roses, metrics including precision (P), recall (R), mean average precision (mAP), and F1-score (F1) were used in the present study. The target confidence threshold was 0.5 and the Intersection over Union (IOU) at the time of testing was 0.5. The P, R, mAP, and IOU are calculated as follows:

$$P=\frac{TP}{TP+FP}$$

$$R=\frac{TP}{TP+FN}$$

$$mAP=\frac{\sum_{i=1}^{k}AP_{i}}{k}$$

$$F1=2\times \frac{\;P\times \;R}{P+R}$$

$$IOU=\frac{Area\;of\;Overlap}{\text{Area of Union}}$$

**Figure 2:**
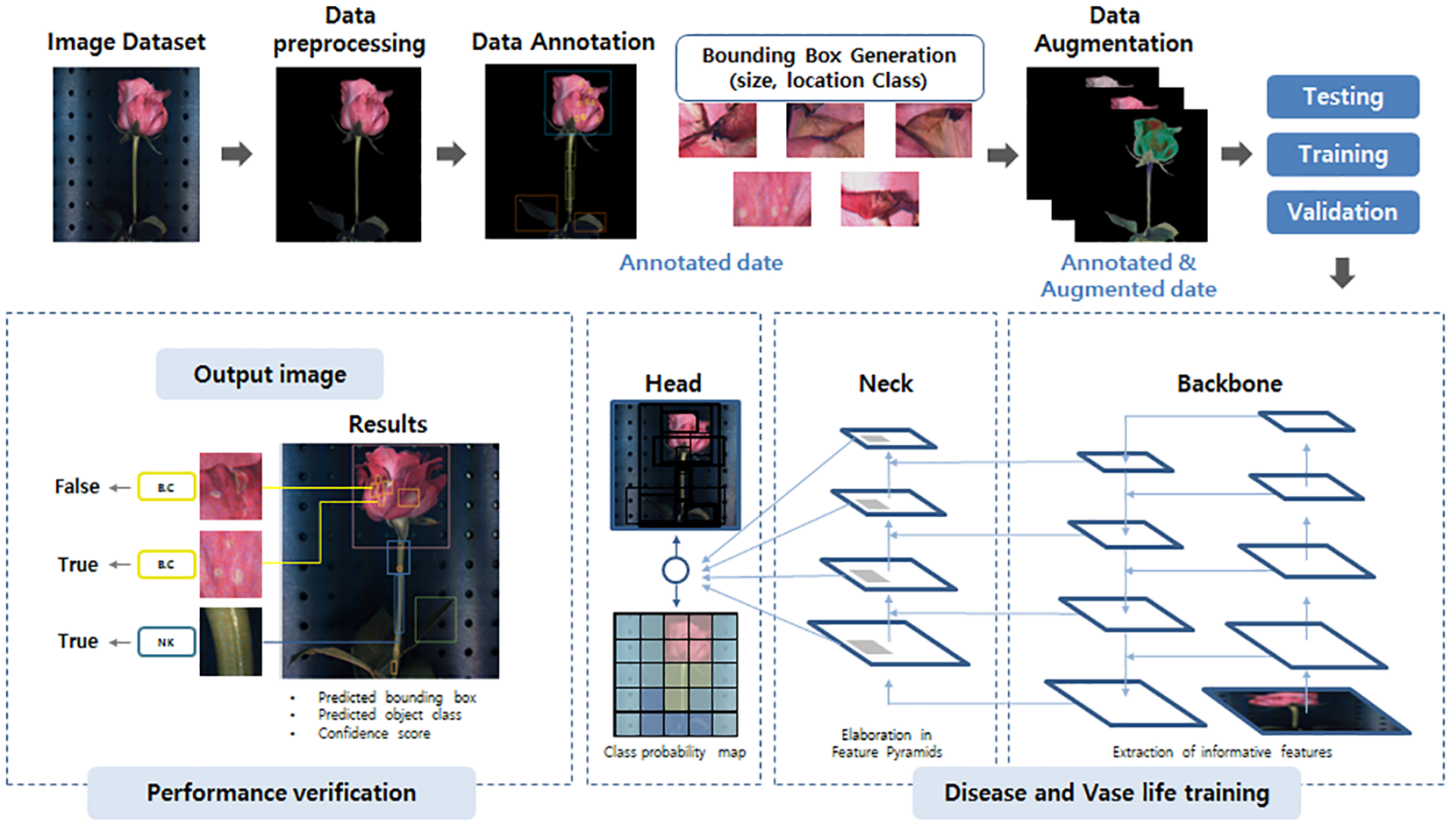
The YOLOv5 network model structure schematic used in this study. The image dataset is first pre-processed, annotated, and undergoes data augmentation to enhance its quality and quantity. The prepared image data is then forwarded to the backbone, the neck, and the head of the model for training and prediction of disease and vase life. Finally, the prediction performance of the models was estimated based on the object-detection values by YOLOv5 system.

Where TP, FP, and FN are the numbers of true positive cases, false positive cases, and false negative cases. The specific network structure of YOLOv5x is shown in **Figure 2**.

To identify the most appropriate image processing model, we also evaluated the performance of two more object detection models: Faster R-CNN and Single Shot Muli-Box Detector (SSD). We utilized the cut rose image dataset, which includes 588 images across 21 categories, showcasing various senescence symptoms. The dataset was partitioned into 70% for training, 15% for validation, and 15% for testing. We tailored the input image sizes to meet the requirements of each model: 640x640 pixels for Faster R-CNN and 512x512 pixels for SSD. All models were implemented using the PyTorch open-source deep learning framework. Each model underwent training with identical hyperparameter settings, including a learning rate set to 0.001, a batch size of 16, and training for a total of 50 epochs.

**Figure 3:**
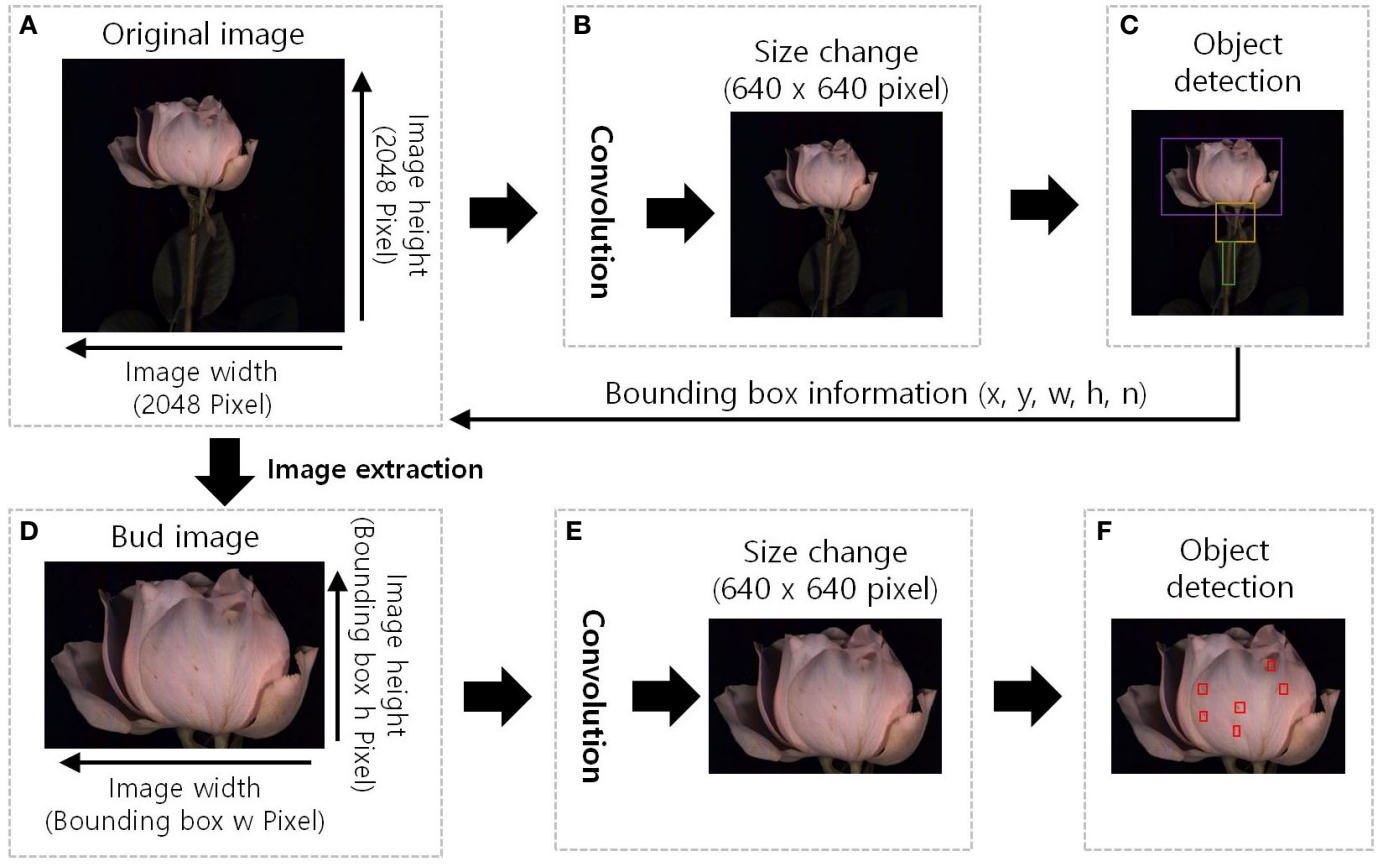
The GMD detection image region extraction pre-processing diagram of the YOLOv5. **(A)**, original HSI with a resolution of 2048 pixels in width and height; **(B)**, the original image was resized to 640x640 pixels, and convolution operations were performed to extract features. **(C)**, detect the object and the bounding box information surrounding the object (x, y, w, h, n) was extracted, x and y: position of the bounding box, w: width, h: height, and n: identification number of the object. **(D)**, bud image is the extracted image of the detected object region, image size is the width and height of the bounding box; **(E)**, resized of 640x640 pixels to standard the input size and additional feature extraction; and **(F)**, red boxes in indicate the detection of disease and wounded spots.

To identify initial disease symptoms and wounded spots, we used an image region extraction pre-processing step using the YOLOv5 object detection algorithm. The flower objects within the images were identified and boxed with a rectangular frame. The objects in the bounding boxes were then precisely cropped and the small spots were detected from the images by the image pre-processing system as shown in **Figure 3**.

A random forest classification model using the object values detected by the YOLOv5x was used to predict the vase life of cut roses. We used a dataset of 200 cut roses corresponding with vase life labels ranging from 1 to 8 d. To optimize the performance of the random forest model, the object values were grouped into feature sets of 1 to 12, based on the importance ranking of the 12 features. The feature sets were constructed by iteratively adding the next most important feature to the previous set until 12 features were included. The dataset was split into training and testing sets using an 80:20 ratio, with stratified sampling to ensure that both sets have a similar distribution of the vase life labels. The random forest model was trained using the training set, with hyperparameters optimized using grid search and cross-validation. The optimized hyperparameters included 100 trees, a maximum depth of 10, and minimum samples required to split a node of 2.

The output of the vase life was classed into two categories as over 5 d (> 5 d) and under 5 d (≤ 5 d) based on the total scores evaluated by gray mold disease (GMD) severity, GMD development weighted value, petal wilting level, and flower opening as shown in **Table 2** and **Supplementary Figure 1**. The scores of quality factors used to predict the vase life of cut roses in **Table 2** were calculated based on the incidence of the vase life terminated factors and GMD disease (**Supplementary Figure 2**). The GMD development weighted value was determined by the growth speed of the disease in petals. The GMD development speed was accelerated by *B. cinerea* inoculation and ethylene treatment and also increased in ‘White Beauty’ compared to ‘All For Love’ (**Supplementary Figure 3**). This evaluation was based on the previous findings showing that ethylene and water stress influenced the progression of GMD in cut roses during transport (Harkema et al., 2013; Ha et al., 2022).

**Table 2:** The scores of quality factors used for to predict the vase life of cut roses using YOLOv5.

GMD severity^x^		GMD weighted value^y^			Petal wilting^z^		Vase life^w^	
Level	Score	Treatment/cultivar		Score	Level	Score	Total score	Output
1	0		None	0	1	0	> 60	≥ 5 D
2	20		*B. cinerea*	20	2	20	< 61	< 5 D
3	40		Ethylene	20	3	40		
4	100	Culti-var	‘All For Love’	0	4	100	< 100	Exclusion
			‘White Beauty’	20				
Total score = 100 – (GMD severity + GMD weighted value + Petal wilting)								

^x^GMD, gray mold disease.

The severity of GMD was evaluated by the area (%) of the disease symptom in rose petals as follows: 1, none; 2, ≤ 3%; 3, 3-10%; and 4, 11-50%.

^y^The weighted value of GMD is the disease developmental speed in rose petals influenced by the treatments and cultivars.

^z^Petal wilting was influenced by water stress and flower opening. It was calculated using four levels as follows: 1, none; 2, slight wilting; 3, moderate wilting; 4, severe wilting.

^w^The total score of vase life was the sum of the scores graded by the quality factors. Vase life of cut roses was classified in two categories: over 5 d (+5D) and under 5 d (-5D) based on the total score. If the total score was ≥ 100, the cut flowers were excluded from the vase life evaluation and classified into the defective group.

### 2.8 Experimental design and statistical analysis

Twenty-five cut roses were used for each treatment. Experiments on vase life and disease evaluation were performed with 10 replicates (one cut flower per replicate). HSI analyses were performed with 6 cut flowers. The remaining 9 cut flowers were used for fungal biomass and gene expression analysis. qRT-PCR analysis was conducted with 3 biological replicates. Data were subjected to analysis of variance (ANOVA) or simple linear regression analysis at *p*< 0.05 using SPSS version 22.0 (IBM, Somers, NY, USA). Data are presented as the mean ± standard error (SE). The experiments were performed twice in both rose cultivars.

## 3 Results

### 3.1 Transport treatments influence vase life, water status, disease infection, and total reflectance of cut roses

**Figure 4:**
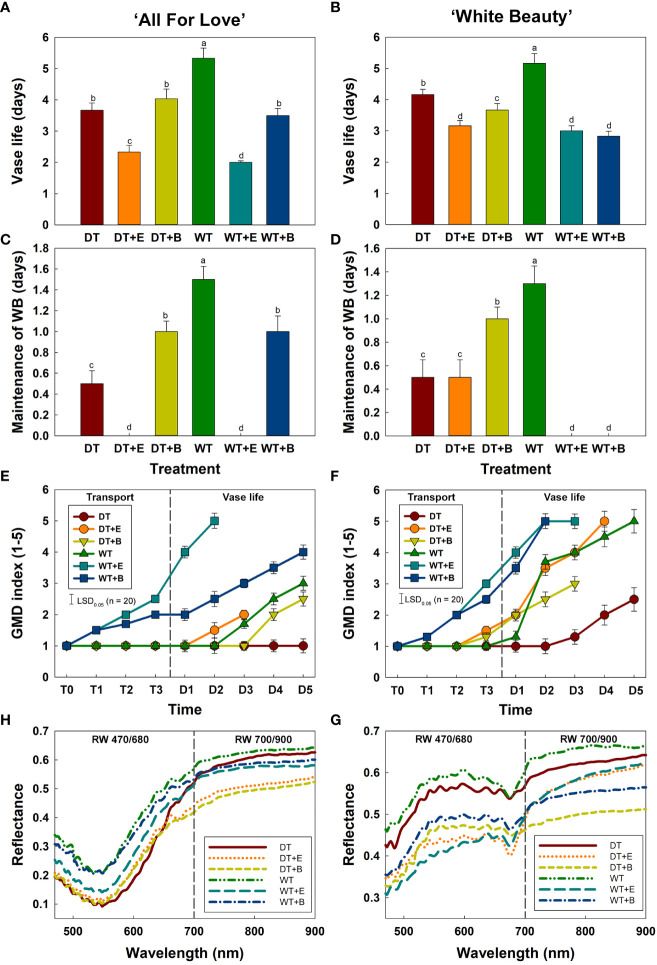
Changes in physiological characteristics of cut roses during transport and vase period. **(A, B)**, vase life; **(C, D)**, maintenance of water balance; **(E, F)**, gray mold disease (GMD) index; **(G, H)**, reflectance of wavelength (RW) in cut roses. DT, dry transport; DT+E, ethylene exposure before DT; DT+B, *B. cinerea* inoculation before DT; WT, wet transport; WT+E, ethylene exposure before WT; WT+B, *B. cinerea* inoculation before WT. GMD was evaluated on days 1-3 of transport (T1–T3) and days 1-5 (D1–D5) of vase period. GMD index was classified into five levels as 1, none; 2, slight symptoms (≤ 3%); 3, moderate symptoms (3-10%); 4, severe symptoms (11-50%); and 5, death of plants (> 50%). RW was detected in cut roses on the first day (D1) of vase period. RW 470/680 and 700/900 indicate the wavelengths from 470 to 680 nm and 700 to 900 nm, respectively. Data are shown as means ± SE (n = 20). Different letters above bars indicate statistically significant differences among treatments at *p* = 0.05 based on Duncan’s multiple range test.

WT treatment extended the vase life of cut roses compared to other treatments (**Figures 4A, B**). WT yielded the longest vase life in both ‘All For Love’ (5.3 d) and ‘White Beauty’ (5.2 d) varieties of cut roses (**Figures 4A, B**). Conversely, DT, ethylene, and *B. cinerea* treatments significantly reduced the vase life of both cultivars (**Figures 4A, B**). Similarly, changes in both cultivars’ capacity to maintain WB mirrored the changes in vase life in response to the different transport treatments. (**Figures 4C, D**).

The first visual symptoms of gray mold disease (GMD) were observed on day 1 (T1) of transport in WT+E and WT+B flowers in both rose cultivars (**Figures 4E, F**). WT+E and WT+B treatments most increased GMD severity in the flower petals during vase periods (**Figures 4E, F**). Although DT reduced the vase life of cut roses, due to water stress caused by an early disruption of water balance, this transport method delayed GMD growth in the flower petals (**Figures 4E, F**). In particular, ‘All For Love’ DT flowers showed no GMD symptoms after transport treatment (**Figure 4E**).

Mean spectral reflectance curves of the cut roses in the wavelength range 470-900 nm obtained using the HSI on the first day (D1) of the vase period are shown in **Figures 4H, G**. The size and shape of flower buds did not influence the reflectance of wavelength in cut rose flowers (**Supplementary Figure 4**). The overall spectral patterns induced by the two treatments were similar for both cultivars. The reflectance of wavelength (RW) in WT flowers was higher than those of other flowers (**Figures 4H, G**), whereas that of DT, DT+E, DT+B, WT+E, and WT+B flowers was relatively low and corresponded with the decline in both vase life and capacity to maintain water balance, as well as and the increase in GMD index (**Figures 4H, G**). The distinct differences in RW in the 470-680 nm range (RW 470/680) in both rose cultivars perhaps show the relation of the spectrums to the susceptibility to the gray mold of the cut flowers (**Figures 4H, G**). Conversely, the differences in RW in the 700-900 nm range (RW 700/900) in both rose cultivars may be correlated with the flower responses to water stress and ethylene (**Figures 4H, G**).

### 3.2 Changes in spectrum curves, fungal growth, and relative expression of genes involved in ethylene biosynthesis, water stress, and senescence of cut roses

**Figure 5:**
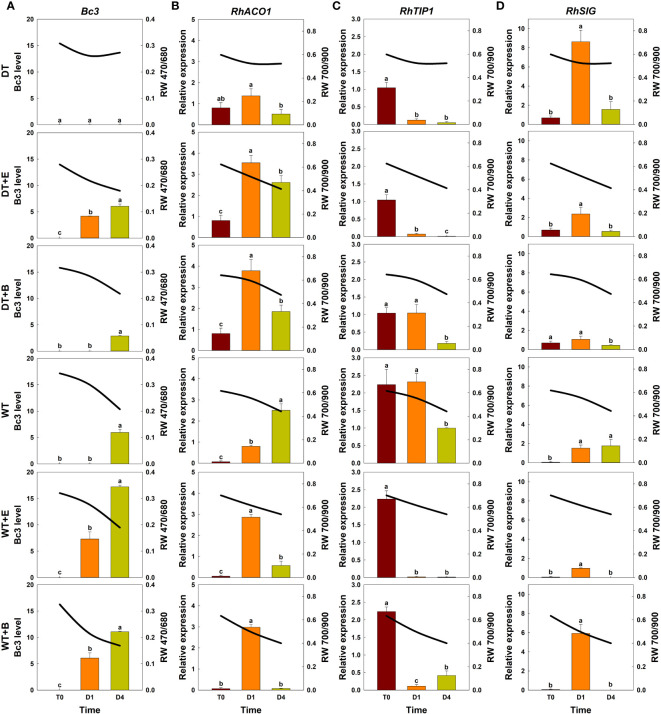
Effect of transport treatments on changes in *B. cinerea* infection level, relative expression of genes related to flower longevity, and average reflectance of wavelength (RW) in ‘All For Love’ cut roses. RW 470/680 and 700/900 indicate the wavelengths of 470 to 680 nm and 700 to 900 nm, respectively. *Bc3*, fungal biomass in rose petals **(A)**; *RhACO1*, ethylene biosynthesis gene **(B)**; *RhTIP*, aquaporin-related gene **(C)**; and *RhSIG*, senescence-induced gene **(D)**. RW, fungal biomass, and gene expression level in cut roses were analyzed on day 0 of transport (T0) and on days 1 (D1) and 4 (D4) of the vase period. DT, dry transport; DT+E, ethylene exposure before DT; DT+B, *B. cinerea* inoculation before DT; WT, wet transport; WT+E, ethylene exposure before WT; WT+B, *B. cinerea* inoculation before WT. The solid line represents the average reflectance of wavelength. The bar charts represent the *Bc3* level, and relative expression of genes related to flower longevity. Data are shown as means ± SE (n = 20 for RW data, 6 for gene expression data). Different letters above bars indicate statistically significant differences among treatments at *p* = 0.05 based on Duncan’s multiple range test.

Changes in spectral reflectance of cut roses in each treatment group (solid lines) were analyzed throughout the transport and vase periods, and the corresponding changes in GMD growth (*Bc3* level) and the relative expression of genes related to ethylene biosynthesis (*RhACO1*), water stress (*RhTIP1*), and senescence induction (*RhSIG*) were also detected in the petals (bar charts) (**Figure 5**).

**Figure 6:**
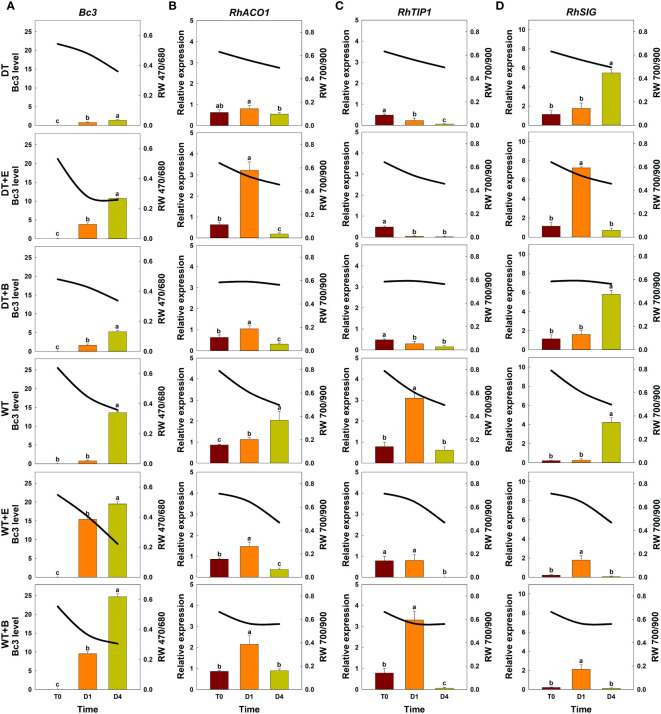
Effect of transport treatments on changes in *B. cinerea* infection level, relative expression of genes related to flower longevity, and average reflectance of wavelength (RW) in ‘White Beauty’ cut roses. RW 470/680 and 700/900 indicate the wavelengths of 470 to 680 nm and 700 to 900 nm, respectively. *Bc3*, fungal biomass in rose petals **(A)**; *RhACO1*, ethylene biosynthesis gene **(B)**; *RhTIP1*, aquaporin-related gene **(C)**; and *RhSIG*, senescence-induced gene **(D)**. RW, fungal biomass, and gene expression level in cut roses were analyzed at day 0 of transport (T0) and at days 1 (D1) and 4 (D4) of the vase period. DT, dry transport; DT+E, ethylene exposure before DT; DT+B, *B. cinerea* inoculation before DT; WT, wet transport; WT+E, ethylene exposure before WT; WT+B, *B. cinerea* inoculation before WT. The solid line represents the average reflectance of wavelength. The bar charts represent the *Bc3* level, and relative expression of genes related to flower longevity. Data are shown as means ± SE (n = 20 for RW data, 6 for gene expression data). Different letters above bars indicate statistically significant differences among treatments at *p* = 0.05 based on Duncan’s multiple range test.

The changes in total spectral reflectance in both rose cultivars after transport treatments are shown in **Supplementary Figure 5**. In the various treatment groups of ‘All For Love’ roses, RW 470/680 during transport (T0) varied in correlation with the level of fungal biomass in petals (**Figure 5A**). Ethylene, *B. cinerea* inoculation, and WT induced high *Bc3* levels rapidly in cut roses while DT reduced *Bc3* levels in rose petals (**Figure 5A**). Thus, RW470/480 in DT+E, DT+B, WT, WT+E, and WT+B flowers rapidly decreased due to *B. cinerea* growth after transport treatments (**Figure 5A**). In contrast, RW 470/480 in DT roses changed only slightly during vase periods (**Figure 5A**). In the case of the ‘White Beauty’, these flowers are particularly susceptible to GMD; thus, the fungal biomass (*Bc3* level) emerged in the petals of all cut roses early at D1 (1^st^ day of the vase period). Consequently, the reduction in RW 470/680 was similar in all flowers (**Figure 6A**).

Ethylene exposure induces higher mRNA levels of the ethylene biosynthesis-related gene *RhACO1* in rose petals (In et al., 2017). Moreover, both ethylene and water stress reduced the expression levels of *RhTIP1*, an aquaporin-related gene (Xue et al., 2009; Ha et al., 2021). These changes induced early senescence symptoms in cut roses by stimulating the expression of senescence-induced genes (**Figures 5B–D**, **6B–D**). In all flowers, a decrease in RW 700/900 corresponded to increased mRNA levels of *RhACO1* and *RhSIG* (a senescence-induced gene) and a decrease in *RhTIP1* expression in petals (**Figures 5B–D**, **6B–D**). At the later stage of the vase period (D4), the death of petal tissues due to GMD or senescence caused a decline in the spectral reflectance of all cut flowers (**Figures 5A–D**, **6A–D**).

**Figure 7:**
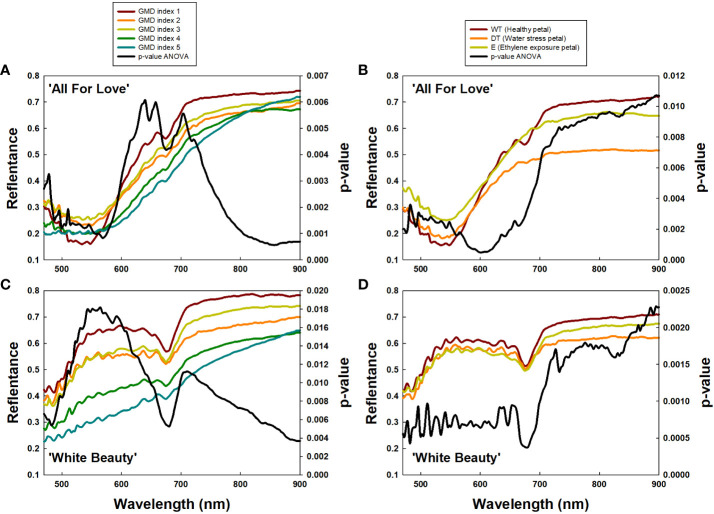
Average reflectance of wavelength (RW) of cut roses based on gray mold disease (GMD) index **(A, C)** and petal wilting level due to water stress or ethylene **(B, C)**, and *p*-values of a one-way ANOVA per RW **(A–D)**. GMD index was classified into five levels as 1, none; 2, slight symptoms (≤ 3%); 3, moderate symptoms (3-10%); 4, severe symptoms (11-50%); and 5, death of plants (> 50%). WT, wet transport; DT, dry transport; and E, ethylene exposure.

To confirm the above results, we extracted the RW 470/680 and RW 700/900 from petals based on GMD index differences (**Figures 7A, C**) and petal wilting level due to water stress or ethylene exposure (**Figures 7B, D**). Subsequently analysis, employing a one-way ANOVA test for each RW, identified RW 600-680 nm in ‘All For Love’ and at RW 500-650 nm in ‘White Beauty’, with notably high *p*-values, closely related to GMD symptom severity (**Figures 7A, C**). Additionally, high *p*-values at RW 700-900 nm indicated distinctions in petal wilting (**Figures 7B, D**). Whereas, *p*-values were low at RW 700/900 and RW 470/680, which are related to GMD severity (**Figures 7A, C**) and petal wilting levels (**Figures 7B, D**). These results indicate that RW 470/680 and RW 700/900 are closely correlated to GMD and other stress conditions respectively in cut rose flowers.

### 3.3 Object detection for GMD using YOLOv5

**Figure 8:**
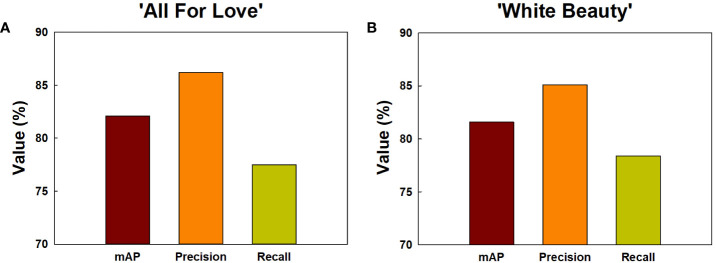
Detection and prediction of gray mold disease in cut roses ‘All For Love’ **(A)** and ‘White Beauty’ **(B)**. The performance of the prediction models by YOLOv5 was evaluated mAP, precision, and recall. mAP, the evaluation index of the detection accuracy; precision, the percentage of true positives (correctly detected objects) out of all the objects that is detected; recall, the percentage of true positives (correctly detected objects) out of all the objects that exist in the dataset.

Among the methods employed for object detection, the YOLOv5 model demonstrated superior accuracy (mAP, precision, and recall) in comparison to the Faster R-CNN and SSD models (**Supplementary Figure 6**). Consequently, the YOLOv5 was chosen for object detection of GMD in cut roses in the present study. The object detection for GMD in cut roses was carried out by YOLOv5x models and the performance of the model was evaluated. The HSI of cut roses was fed into the YOLOv5x model which was trained to identify the presence of GMD in petals. The model effectively detected small instances of GMD in rose petals (**Figure 1C**), demonstrating that YOLOv5x can predict the disease emergence at the early stages of the disease infection. The mAP represents the evaluation index of disease detection accuracy. In this study, mAP value was relatively high (82.1%) in ‘All For Love’ flowers (**Figure 8A**). The precision (86.2%) and recall (77.5%) values achieved by the model were also high in ‘All For Love’ flowers (**Figure 8A**). Whereas, the performance of the YOLOv5 model for ‘White Beauty’ flowers was slightly lower (mAP, 81.6%; precision, 85.1%; and recall, 78.4%) (**Figure 8B**). Nevertheless, these values were enough high and better than those of the prediction based on petal wilting levels (**Supplementary Figure 7**).

### 3.4 Prediction of vase life of cut roses using YOLOv5

**Figure 9:**
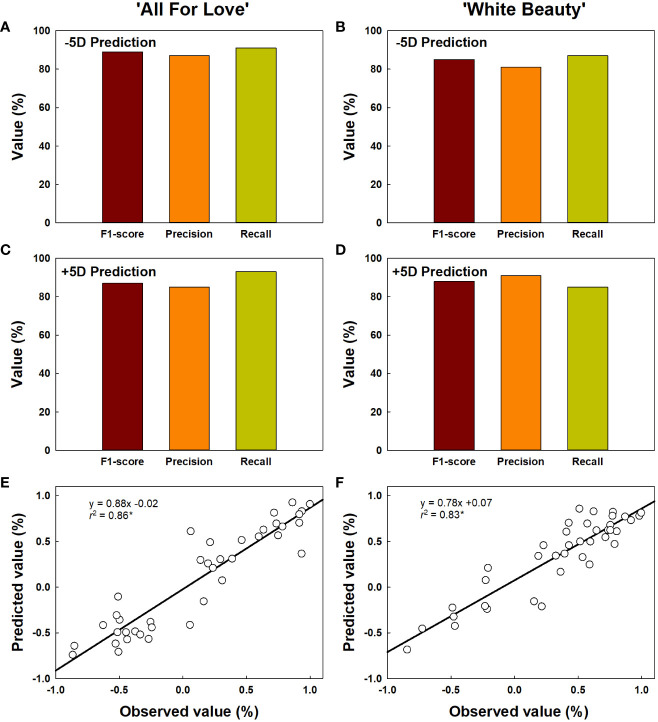
Prediction of vase life of cut roses ‘All For Love’ **(A, C)** and ‘White Beauty’ **(B, D)**. The performance of the prediction model by random forest was evaluated F1-score, precision, and recall. F1-score, the harmonic mean of precision; precision, the percentage of true positives (correctly detected objects) out of all the objects that is detected; recall, the percentage of true positives (correctly detected objects) out of all the objects that exist in the dataset. The accuracy of vase life prediction **(E, F)** by YOLOv5 and random forest. The vase life of cut roses were classed into two categories as over 5 d (+5D) and under 5 d (-5D) based on the scores graded by the quality factors in **Table 2**. The negative (-1.0-0) and positive (0.0-1.0) values by the linear regression analysis respectively indicate the probability that the vase life is -5D or +5D. Asterisk (*) represents a significant difference at *p* = 0.05 (n = 40).

The classification for vase life in cut roses was carried out by random forest models and the performance of the model was evaluated. The vase life of cut roses was trained into two categories as under 5 d (-5D) and over 5 d (+5D) based on the scores graded by the quality factors presented in **Table 2**. In this study, we evaluated the classification performance of the random forest algorithm in both cultivars. In ‘All For Love’ rose flowers, in the -5D case, the model displayed an F1 score of 89%, precision of 87%, and recall of 91% (**Figure 9A**). In contrast, in the +5D case, the performance was slightly lower (F1, 87%; precision, 85%; and Recall, 93%) (**Figure 9C**). In ‘White Beauty’ rose flowers, in the -5D case, the model yielded an F1 score of 85%, precision of 81%, and recall of 87% (**Figure 9B**). However, in the +5D case, the performance was slightly higher, with an F1 score of 88%, precision of 91%, and recall of 85% (**Figure 9D**).

The vase life prediction model was developed using YOLOv5x based on the detection of petal conditions (**Figures 9E, F**). As a result, the scatter plots showed a strong correlation between the predicted value and the observed value of the vase life evaluation (*r*^2 ^= 0.86 in ‘All For Love’ and 0.83 in ‘White Beauty’) (**Figures 9E, F**). This result indicates that the YOLOv5 model achieves a strong capacity for the vase life prediction of cut flowers by analyzing the large size of the complicated data obtained HSI.

## 4 Discussion

Postharvest conditions, such as dry transport, ethylene or high density of mold spores have been observed to decrease the longevity of the cut roses (Harkema et al., 2013; Ha et al., 2021; Ha et al., 2022). In this study, dry transport, ethylene exposure, and increased *B. cinerea* spore (induced by ethylene exposure and fungal conidial inoculation during transport simulation) significantly reduced vase life and positive water balance of cut roses. Dry transport, a practice involving storing cut flowers without water to facilitate transportation or control *B. cinerea* growth, can lead to dehydration and reduced vase life of cut flowers (Macnish et al., 2009; Harkema et al., 2013; Fanourakis et al., 2022). Ethylene exposure accelerates the senescence process of cut roses, leading to premature wilting, petal abscission, and overall reduced vase life (Macnish et al., 2010; In et al., 2017). During transportation, contact with *B. cinerea* spores or storage in conditions conductive to fungal growth can lead to infection, resulting in necrotic lesions and decay, ultimately reducing vase life of cut flowers (Ha et al., 2022).

Hyperspectral imaging is a non-contact method that analyses a wide range of light spectrums by scanning objects with hyperspectral cameras (Lowe et al., 2017; Cao et al., 2022; Xiang et al., 2022). The reflectance of light from plants at different wavelengths can be used to obtain information about various plant statuses and conditions (Sun J et al., 2021; Sukhova and Yudina, 2022). In this study, HSI of cut roses was used to observe distinct wavelength ranges of plants in various physiological states, such as GMD infection, water stress response, and senescence induction. The spectral reflectance at 470-680 nm was found to be strongly related to *B. cinerea* infection in the rose petals. The reflectance in this wavelength range is mainly affected by the absorption spectra of pigments in the leaves or petals of the plants (Rolfe and Scholes, 2010). *B. cinerea* infection would change the content and distribution of the pigment in the petals leading to changes in the spectral reflectance (López-López et al., 2016). While water stress causes changes in the water content of plant tissues which in turn affects the reflectance of light in the wavelength range of 700-900 nm (Elvanidi et al., 2018). Similarly, the reflectance at 700-900 nm was highly correlated to the petal wilting levels due to water stress after dry-transport or ethylene in cut flowers. Our results are consistent with those of previous studies showing that the reflectance at 400-680 nm is related to disease infection and the reflectance at higher 700 nm is sensitive to vegetation stress or water stress (Thenkabail et al., 2004; Köksal, 2011; Cao et al., 2022).

The YLOLv5x model was established to predict the potential incidence rate of GMD and the vase life of cut roses based on HSI data. We used the mAP@0.5 indicator to measure the prediction model’s overall performance on the training test. The resulting mAP@0.5 value of the YOLOv5x model was approximately 80% in ‘All For Love’ roses, indicating that the model has a high prediction accuracy and can detect even small traces of fungal at early stages of disease development in rose petals. In previous studies, a similar detection performance was observed when YOLOv5 was used to predict powdery mildew disease and anthracnose in rubber plants (Chen et al., 2022). Our results also showed that the precision (78.6%) and recall (80.5%) values achieved from the model were also relatively high for ‘All For Love’ flowers, indicating that the model has a low chance of wrong detection (Qi et al., 2022). However, the disease detection performance was slightly lower for ‘White Beauty’ cultivar, possibly due to color similarity between white petals and GMD symptoms (Del Valle et al., 2018; Kasajima, 2019; Jiang et al., 2021).

Previously, the vase life prediction models in cut roses were developed by using the combination of machine learning and thermal imaging based on the different temperatures of petals among flower blooming stages (Choi and Lee, 2020). Evaluation of the flower quality of cut roses using a four-dimensional deep learning method was also studied based on the flower maturing status (Sun X et al., 2021). Despite the relatively good prediction accuracy of the models, an application of these techniques is difficult because the performance of the models is suitable only in limited conditions. In this study, the YOLOv5x models performed the vase life prediction well based on the detection of the flower states under different stress conditions and transportation methods. The results revealed that the models developed here are outstanding in the accuracy of the vase life prediction, consequently, applicability to the flower industry.

However, our model was developed using only two rose cultivars, thus further validation of the model with a larger dataset from various cultivars and environmental conditions is required to establish its general applicability. Furthermore, optimization of the YOLOv5 model, considering factors such as dataset size (Fang et al., 2021; Doherty et al., 2022) and computational resources (Junior and Ulson, 2021; Li et al., 2022), is crucial for improved performance and broader applicability.

## 5 Conclusion

In conclusion, our results have demonstrated the potential use of deep learning algorithms for detecting GMD and predicting the vase life of cut roses based on hyperspectral images of flower bud states. The finding from this study revealed that the spectral reflectance of 470 to 680 nm and 700 to 900 nm was closely related to GMD and plant physiological conditions, respectively in cut roses. The YOLOv5 model precisely detected and classified *B. cinerea* infection with high precision. The model also showed high predictive accuracy in evaluating the vase life of cut roses based on extensive image processing. With some modifications, the vase life prediction models developed in this study could be effective tools for constructing a flower longevity guarantee system for the flower industry.

## Data availability statement

The datasets presented in this study can be found in online repositories. The names of the repository/repositories and accession number(s) can be found in the article/**Supplementary Material**.

## Author contributions

Y-TK: Investigation, Data curation, Formal analysis, Software, Validation, Visualization, Writing – original draft. SH: Data curation, Formal analysis, Investigation, Writing – original draft. B-CI: Investigation, Conceptualization, Funding acquisition, Methodology, Project administration, Resources, Supervision, Writing – review & editing.

## References

[B1] Ahmad I. Yang Y. Yue Y. Ye C. Hassan M. Cheng X.. (2022). Deep learning based detector YOLOv5 for identifying insect pests. Appl. Sci. 12, 10167. doi: 10.3390/app121910167

[B2] Amadi-Majd M. Nejad A. R. Mousavi-Fard S. Fanourakis D. (2021). Deionized water as vase solution prolongs flower bud opening and vase life in carnation and rose through sustaining an improved water balance. Eur. J. Hortic. Sci. 86, 682–693. doi: 10.17660/eJHS.2021/86.6.12

[B3] Behmann J. Steinrücken J. Plümer L. (2014). Detection of early plant stress responses in hyperspectral images. J. Photogramm. Remote Sens. 93, 98–111. doi: 10.1016/j.isprsjprs.2014.03.016

[B4] Bulgari R. Petrini A. Cocetta G. Nicoletto C. Ertani A. Sambo P.. (2021). The impact of COVID-19 on horticulture: Critical issues and opportunities derived from an unexpected occurrence. Horticulturae 7, 124. doi: 10.3390/horticulturae7060124

[B5] Cao Y. Yuan P. Xu H. Martínez-Ortega J. F. Feng J. Zhai Z. (2022). Detecting asymptomatic infections of rice bacterial leaf blight using hyperspectral imaging and 3-dimensional convolutional neural network with spectral dilated convolution. Front. Plant Sci. 13. doi: 10.3389/fpls.2022.963170 PMC9328758 35909723

[B6] Cape J. N. (2003). Effects of airborne volatile organic compounds on plants. Environ. Pollut. 122, 145–157. doi: 10.1016/s0269-7491(02)00273-7 12535603

[B7] Chagué V. Danit L.V. Siewers V. Schulze-Gronover C. Tudzynski P. Tudzynski B.. (2006). Ethylene sensing and gene activation in Botrytis cinerea: a missing link in ethylene regulation of fungus-plant interactions? Mol. Plant. Microbe. Interact. 19, 33–42. doi: 10.1094/mpmi-19-0033 16404951

[B8] Chang C. Bleecker A. B. (2004). Ethylene biology. More than a gas. Plant Physiol. 136, 2895–2899. doi: 10.1104/pp.104.900122 15489282 PMC523351

[B9] Chen Z. Wu R. Lin Y. Li C. Chen S. Yuan Z.. (2022). Plant disease recognition model based on improved YOLOv5. Agronomy 12, 365. doi: 10.3390/agronomy12020365

[B10] Choi S. Y. Lee A. K. (2020). Development of a cut rose longevity prediction model using thermography and machine learning. Hortic. Sci. Technol. 38, 675–685. doi: 10.7235/HORT.20200061

[B11] Cravero A. Pardo S. Sepúlveda S. Muñoz L. (2022). Challenges to use machine learning in agricultural big data: a systematic literature review. Agronomy 12, 748. doi: 10.3390/agronomy12030748

[B12] Del Valle J. C. Gallardo-López A. Buide M. L. Whittall J. B. Narbona E. (2018). Digital photography provides a fast, reliable, and noninvasive method to estimate anthocyanin pigment concentration in reproductive and vegetative plant tissues. Eco. Evol. 8, 3064–3076. doi: 10.1002/ece3.3804 PMC5869271 29607006

[B13] Doherty J. Gardiner B. Kerr E. Siddique N. Manvi S. S. (2022). "Comparative study of activation functions and their impact on the YOLOv5 object detection model." in International Conference on Pattern Recognition and Artificial Intelligence (Cham: Springer International Publishing), 40–52.

[B14] Elvanidi A. Katsoulas N. Ferentinos K. P. Bartzanas T. Kittas C. (2018). Hyperspectral machine vision as a tool for water stress severity assessment in soilless tomato crop. Biosyst. Eng. 165, 25–35. doi: 10.1016/j.biosystemseng.2017.11.002

[B15] Fang Y. Guo X. Chen K. Zhou Z. Ye Q. (2021). Accurate and automated detection of surface knots on sawn timbers using YOLO-V5 model. Bioresources 16, 5390–5406. doi: 10.15376/biores.16.3.5390-5406

[B16] Fanourakis D. Giday H. Li T. Kambourakis E. Ligoxigakis E. K. Papadimitriou M.. (2016). Antitranspirant compounds alleviate the mild-desiccation-induced reduction of vase life in cut roses. Postharvest Biol. Technol. 117, 110–117. doi: 10.1016/j.postharvbio.2016.02.007

[B17] Fanourakis D. Paparakis V. M. Psyllakis E. Tzanakakis V. A. Nektarios P. (2022). The role of water relations and oxidative stress in the vase life response to prolonged storage: A case sttudy in chrysanthemum. Agriculture. 12 (2), 185. doi: 10.3390/agriculture12020185

[B18] Fanourakis D. Pieruschka R. Savvides A. Macnish A. J. Sarlikioti V. Woltering E. J. (2013). Sources of vase life variation in cut roses: A review. Postharvest Biol. Technol. 78, 1–15. doi: 10.1016/j.postharvbio.2012.12.001

[B19] Fanourakis D. Velez-Ramirez A. I. In B. C. Barendse H. Van Meeteren U. Woltering E. J. (2015). A Survey of preharvest conditions affecting the regulation of water loss during vase life. Acta Hortic. 1064, 195–204. doi: 10.17660/ActaHortic.2015.1064.22

[B20] Friedman H. Agami O. Vinokur Y. Droby S. Cohen L. Refaeli G.. (2010). Characterization of yield, sensitivity to *Botrytis cinerea* and antioxidant content of several rose species suitable for edible flowers. Sci. Hortic. 123, 395–401. doi: 10.1016/j.scienta.2009.09.019

[B21] Gabellini S. Scaramuzzi S. (2022). Evolving consumption trends, marketing strategies, and governance settings in ornamental horticulture: A grey literature review. Horticulturae 8, 234. doi: 10.3390/horticulturae8030234

[B22] Guo Y. Liu Y. Oerlemans A. Lao S. Wu S. Lew M. S. (2016). Deep learning for visual understanding: A review. Neurocomputing 187, 27–48. doi: 10.1016/j.neucom.2015.09.116

[B23] Ha S. T. T. Kim Y.-T. Yeam I. Choi H. W. In B.-C. (2022). Molecular dissection of rose and *Botrytis cinerea* pathosystems affected by ethylene. Postharvest Biol. Technol. 194, 112104. doi: 10.1016/j.postharvbio.2022.112104

[B24] Ha S. T. T. Nguyen T. K. Lim J. H. (2021). Effects of air-exposure time on water relations, longevity, and aquaporin-related gene expression of cut roses. Hortic. Environ. Biotechnol. 62, 63–75. doi: 10.1007/s13580-020-00302-1

[B25] Harkema H. Mensink M. G. J. Somhorst D. P. M. Pedreschi R. P. Westra E. H. (2013). Reduction of *Botrytis cinerea* incidence in cut roses (*Rosa hybrida* L.) during long term transport in dry conditions. Postharvest Biol. Technol. 76, 135–138. doi: 10.1016/j.postharvbio.2012.10.003

[B26] In B.-C. Ha S. T. T. Lee Y. S. Lim J. H. (2017). Relationships between the longevity, water relations, ethylene sensitivity, and gene expression of cut roses. Postharvest Biol. Technol. 131, 74–83. doi: 10.1016/j.postharvbio.2017.05.003

[B27] In B.-C. Inamoto K. Doi M. (2009). A neural network technique to develop a vase life prediction model of cut roses. Postharvest Biol.Technol. 52, 273–278. doi: 10.1016/j.postharvbio.2009.01.001

[B28] In B.-C. Inamoto K. Doi M. Park S.-A. (2016a). Using thermography to estimate leaf transpiration rates in cut roses for the development of vase life prediction models. Hortic. Environ. Biotechnol. 57, 53–60. doi: 10.1007/s13580-016-0117-6

[B29] In B.-C. Lee J.-H. Lee A.-K. Lim J. H. (2016b). Conditions during export affect the potential vase life of cut roses (*Rosa hybrida* L.). Hortic. Environ. Biotechnol. 57, 504–510. doi: 10.1007/s13580-016-1119-0

[B30] In B.-C. Lim J. H. (2018). Potential vase life of cut roses: Seasonal variation and relationships with growth conditions, phenotypes, and gene expressions. Postharvest Biol. Technol. 135, 93–103. doi: 10.1016/j.postharvbio.2017.09.006

[B31] Ji Y. Sun L. Li Y. Ye D. (2019). Detection of bruised potatoes using hyperspectral imaging technique based on discrete wavelet transform. Infrared Phys. Technol. 103, 103054. doi: 10.1016/j.infrared.2019.10305

[B32] Jiang Q. Wu G. Tian C. Li N. Yang H. Bai Y.. (2021). Hyperspectral imaging for early identification of strawberry leaves diseases with machine learning and spectral fingerprint features. Infrared Phys. Technol. 118, 103898. doi: 10.1016/j.infrared.2021.10389

[B33] Junior L. C. M. Ulson J. (2021). “Real time weed detection using computer vision and deep learning,” in 2021 14th IEEE International Conference on Industry Applications (INDUSCON), São Paulo, Brazil, pp. 1131–1137. doi: 10.1109/INDUSCON51756.2021.9529761

[B34] Kamilaris A. Prenafeta-Boldú F. X. (2018). Deep learning in agriculture: A survey. Comput. Electron. Agric. 147, 70–90. doi: 10.1016/j.compag.2018.02.016

[B35] Kasajima I. (2019). Measuring plant colors. Plant Biotechnol. 36, 63–75. doi: 10.5511/plantbiotechnology.19.0322a PMC6847779 31768106

[B36] Köksal E. S. (2011). Hyperspectral reflectance data processing through cluster and principal component analysis for estimating irrigation and yield related indicators. Agric. Water Manage. 98, 1317–1328. doi: 10.1016/j.agwat.2011.03.014

[B37] Lay L. Lee H. S. Tayade R. Ghimire A. Chung Y. S. Yoon Y.. (2023). Evaluation of soybean wildfire prediction via hyperspectral imaging. Plants 12, 901. doi: 10.3390/plants12040901 36840248 PMC9967622

[B38] LeCun Y. Bengio Y. Hinton G. (2015). Deep learning. Nature 521, 436–444. doi: 10.1038/nature14539 26017442

[B39] Li J. Qiao Y. Liu S. Zhang J. Yang Z. Wang M. (2022). An improved YOLOv5-based vegetable disease detection method. Comput. Electron. Agr. 202, 107345. doi: 10.1016/j.compag.2022.107345

[B40] Liu D. Zeng X. A. Sun D. W. (2015). Recent developments and applications of hyperspectral imaging for quality evaluation of agricultural products: a review. Crit. Rev. Food. Sci. Nutr. 55, 1744–1757. doi: 10.1080/10408398.2013.777020 24915395

[B41] López-López M. Calderón R. González-Dugo V. Zarco-Tejada P. J. Fereres E. (2016). Early detection and quantification of almond red leaf blotch using high-resolution hyperspectral and thermal imagery. Remote Sens. 8, 276. doi: 10.3390/rs8040276

[B42] Lowe A. Harrison N. French A. P. (2017). Hyperspectral image analysis techniques for the detection and classification of the early onset of plant disease and stress. Plant Methods 13, 80. doi: 10.1186/s13007-017-0233-z 29051772 PMC5634902

[B43] Macnish A. J. De Theije A. Reid M. S. Jiang C. Z. (2009). An alternative postharvest handing strategy for cut flowers-dry handling after harvest. Acta Hortic. 847, 215–222. doi: 10.17660/ActaHortic.2009.847.27

[B44] Macnish A. J. Leonard R. T. Borda A. M. Nell T. A. (2010). Genotypic variation in the postharvest performance and ethylene sensitivity of cut rose flowers. J. Am. Soc Hortic. Sci. 45, 790–796. doi: 10.21273/HORTSCI.45.5.790

[B45] Martínez-Romero D. Guillén F. Castillo S. Zapata P. J. Serrano M. Valero D. (2009). Development of a carbon-heat hybrid ethylene scrubber for fresh horticultural produce storage purposes. Postharvest Biol. Technol. 51, 200–205. doi: 10.1016/j.postharvestbio.2008.07.013

[B46] Mo C. Kim G. Lim J. Kim M. S. Cho H. Cho B.-K. (2015). Detection of lettuce discoloration using hyperspectral reflectance imaging. Sensors 15, 29511–29534. doi: 10.3390/s151129511 26610510 PMC4701346

[B47] Nasiri A. Taheri-Garavand A. Fanourakis D. Zhang Y.-D. Nikoloudakis N. (2021). Automated grapevine cultivar identification via leaf imaging and deep convolutional neural networks: A proof-of-concept study employing primary Iranian varieties. Plants 10, 1628. doi: 10.3390/plants10081628 34451673 PMC8399703

[B48] Qi J. Liu X. Liu K. Xu F. Guo H. Tian X.. (2022). An improved YOLOv5 model based on visual attention mechanism: Application to recognition of tomato virus disease. Comput. Electron. Agr. 194, 106780. doi: 10.1016/j.compag.2022.106780

[B49] Rahman A. Kandpal L. M. Lohumi S. Kim M. S. Lee H. Mo C.. (2017). Nondestructive estimation of moisture content, pH and soluble solid contents in intact tomatoes using hyperspectral imaging. Appl. Sci. 7, 109. doi: 10.3390/app7010109

[B50] Ramamoorthy P. Samiappan S. Wubben M. J. Brooks J. P. Shrestha A. Panda R. M.. (2022). Hyperspectral reflectance and machine learning approaches for the detection of drought and root-knot nematode infestation in cotton. Remote Sens. 14, 4021. doi: 10.3390/rs14164021

[B51] Redmon J. Divvala S. Girshick R. Farhadi A. (2016). “You Only Look Once: unified, real-time object detection,” in 2016 IEEE Conference on Computer Vision and Pattern Recognition (CVPR), Las Vegas, NV, USA, pp. 779–788. doi: 10.1109/CVPR.2016.91

[B52] Reid M. S. Mokhtari M. Lieth J. H. van Doorn W. G. Evans R. Y. (1996). Modeling the postharvest life of cut roses. Acta Hortic. 424, 137–144. doi: 10.17660/ActaHortic.1996.424.24

[B53] Rolfe S. A. Scholes J. D. (2010). Chlorophyll fluorescence imaging of plant–pathogen interactions. Protoplasma 247, 163–175. doi: 10.1007/s00709-010-0203-z 20814703

[B54] Staby G. L. Cunningham M. S. (1980). Predicting longevity of carnations to reduce postharvest shrinkage. Ohio Rep. 65, 54–55.

[B55] Stead A. D. Gay A. Taylor J. Ougham H. Wagstaff C. Rogers H. J. (2018). Hyperspectral imaging as a means to assess quality issues of cut flowers. Acta Hortic. 1263, 359–366. doi: 10.17660/ActaHortic.2009.1263.47

[B56] Suarez M. B. Walsh K. Boonham N. O'Neill T. Pearson S. Barker I.. (2005). Development of real-time PCR (TaqMan) assays for the detection and quantification of *Botrytis cinerea* in planta. Plant Physiol. Biochem. 43, 890–899. doi: 10.1016/j.plaphy.2005.07.003 16198585

[B57] Sukhova E. Yudina L. (2022). Modified photochemical reflectance indices as new tool for revealing Influence of drought and heat on pea and wheat plants. Plants 11, 1380. doi: 10.3390/plants11101308 35631733 PMC9147454

[B58] Sun X. Li Z. Zhu T. Ni C. (2021). Four-dimension deep learning method for flower quality grading with depth information. Electronics 10, 2353. doi: 10.3390/electronics10192353

[B59] Sun J. Yang L. Yang X. Wei J. Li L. Guo E.. (2021). Using spectral reflectance to estimate the leaf chlorophyll content of maize inoculated with *Arbuscular Mycorrhizal* fungi under water stress. Front. Plant Sci. 12. doi: 10.3389/fpls.2021.646173 PMC8193845 34122471

[B60] Susič N. Žibrat U. Širca S. Strajnar P. Razinger J. Knapič M.. (2018). Discrimination between abiotic and biotic drought stress in tomatoes using hyperspectral imaging. Sens. Actuators B Chem. 273, 842–852. doi: 10.1016/j.snb.2018.06.121

[B61] Taghizadeh M. Gowen A. A. O’donnell C. P. (2011). The potential of visible-near infrared hyperspectral imaging to discriminate between casing soil, enzymatic browning and undamaged tissue on mushroom (*Agaricus bisporus*) surfaces. Comput. Electron. Agr. 77, 74–80. doi: 10.1016/j.compag.2011.03.010

[B62] Taheri-Garavand A. Mumivand H. Fanourakis D. Fatahi S. Taghipour S. (2021). An artificial neural network approach for non-invasive estimation of essential oil content and composition through considering drying processing factors: A case study in Mentha aquatica. Ind. Crop Prod. 171, 113985. doi: 10.1016/j.indcrop.2021.113985

[B63] Thenkabail P. S. Enclona E. A. Ashton M. S. van der Meer B. (2004). Accuracy assessments of hyperspectral waveband performance for vegetation analysis applications. Remote Sens. Environ. 91, 354–376. doi: 10.1016/j.rse.2004.03.013

[B64] Tian H. Wang T. Liu Y. Qiao X. Li Y. (2020). Computer vision technology in agricultural automation —A review. Inf. Process. Agric. 7, 1–19. doi: 10.1016/j.inpa.2019.09.006

[B65] Tromp S.-O. van der Sman R. G. M. Vollebregt H. M. Woltering E. J. (2012). On the prediction of the remaining vase life of cut roses. Postharvest Biol. Technol. 70, 42–50. doi: 10.1016/j.postharvbio.2012.04.003

[B66] VBN (2014). Evaluation cards for Rosa (The Netherlands: FloraHollandAalsmeer).

[B67] Vehniwal S. S. Abbey L. (2019). Cut flower vase life – influential factors, metabolism, and organic formulation. Horticult. Int. J. 3, 275–281. doi: 10.15406/hij.2019.03.00142

[B68] Veys C. Chatziavgerinos F. Alsuwaidi A. Hibbert J. Hansen M. Bernotas G.. (2019). Multispectral imaging for presymptomatic analysis of light leaf spot in oilseed rape. Plant Methods 15, 4. doi: 10.1186/s13007-019-0389-9 30697329 PMC6345015

[B69] Wang K. L.-C. Li H. Ecker J. R. (2002). Ethylene biosynthesis and signaling networks. Plant Cell 14, S131–S151. doi: 10.1105/tpc.001768 12045274 PMC151252

[B70] Wieme J. Mollazade K. Malounas I. Zude-Sasse M. Zhao M. Gowen A.. (2022). Application of hyperspectral imaging systems and artificial intelligence for quality assessment of fruit, vegetables and mushrooms: A review. Biosyst. Eng. 222, 156–176. doi: 10.1016/j.biosystemseng.2022.07.013

[B71] Williamson B. Tudzynski B. Tudzynski P. Van Kan J. A. (2007). *Botrytis cinerea*: the cause of grey mould disease. Mol. Plant Pathol. 8, 561–580. doi: 10.1111/j.1364-3703.2007.00417.x 20507522

[B72] Xiang Y. Chen Q. Su Z. Zhang L. Chen Z. Zhou G.. (2022). Deep learning and hyperspectral images based tomato soluble solids content and firmness estimation. Front. Plant Sci. 13. doi: 10.3389/fpls.2022.860656 PMC9108868 35586212

[B73] Xue J. Yang F. Gao J. (2009). Isolation of *Rh-TIP1;1*, an aquaporin gene and its expression in rose flowers in response to ethylene and water deficit. Postharvest Biol. Technol. 51, 407–413. doi: 10.1016/j.postharvbio.2008.08.011

[B74] Yao J. Qi J. Zhang J. Shao H. Yang J. Li X. (2021). A real-time detection algorithm for kiwi fruit defects based on YOLOv5. Electronics 10, 1711. doi: 10.3390/electronics10141711

[B75] Zhang C. Guo C. Liu F. Kong W. He Y. Lou B. (2016). Hyperspectral imaging analysis for ripeness evaluation of strawberry with support vector machine. J. Food Eng. 179, 11–18. doi: 10.1016/j.jfoodeng.2016.01.002

[B76] Zhang Y. He S. Wa S. Zong Z. Liu Y. (2021). Using generative module and pruning inference for the fast and accurate detection of apple flower in natural environments. Information 12, 495. doi: 10.3390/info12120495

