# Supplementary material for: Development of a longevity prediction model for cut roses using hyperspectral imaging and a convolutional neural network
Source: Front Plant Sci. 2024 Jan 10;14:1296473. doi: 10.3389/fpls.2023.1296473 (PMC10809400; doi:10.3389/fpls.2023.1296473)
Supplement: Supplementary file 1 [file DataSheet_1.docx]

***Supplementary Material***

**Supplementary Figures**


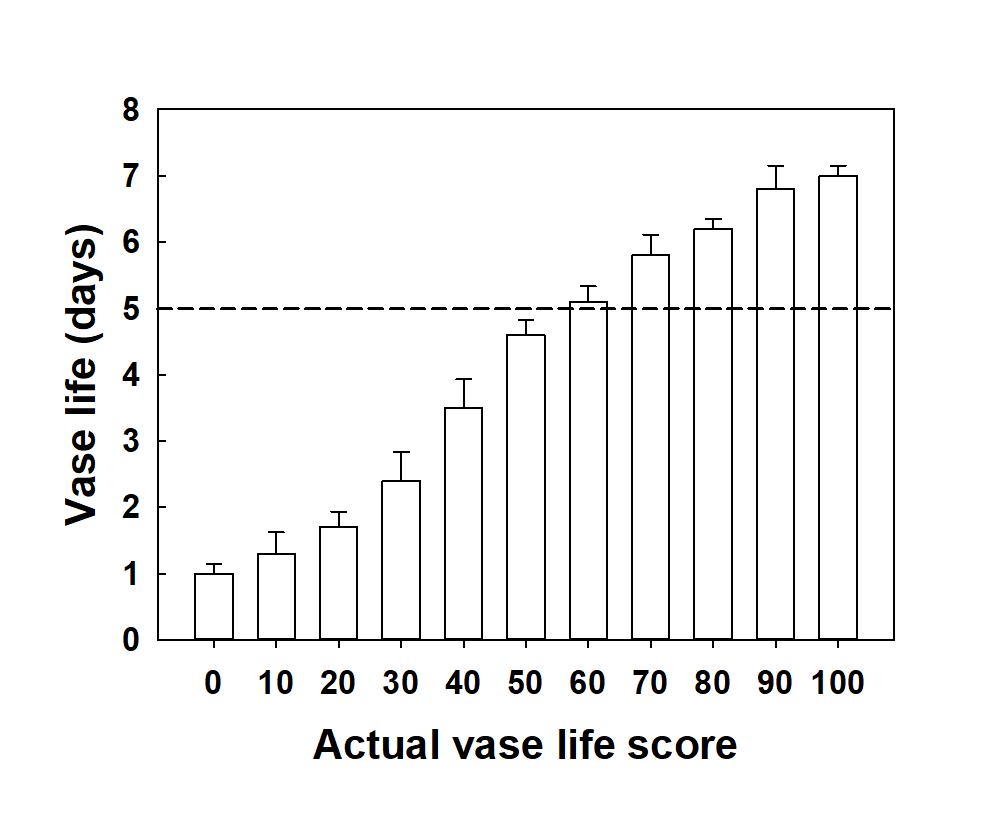


**Supplementary Figure S1.** Vase life score based on the calculation from the score of quality factors used to predict the vase life. The output of the vase life was classed into two categories: over 5 d (+5D) and under 5 d (-5D) based on the total scores evaluated by gray mold disease (GMD) severity, GMD development weighted value, and petal wilting level as shown in Table 2.


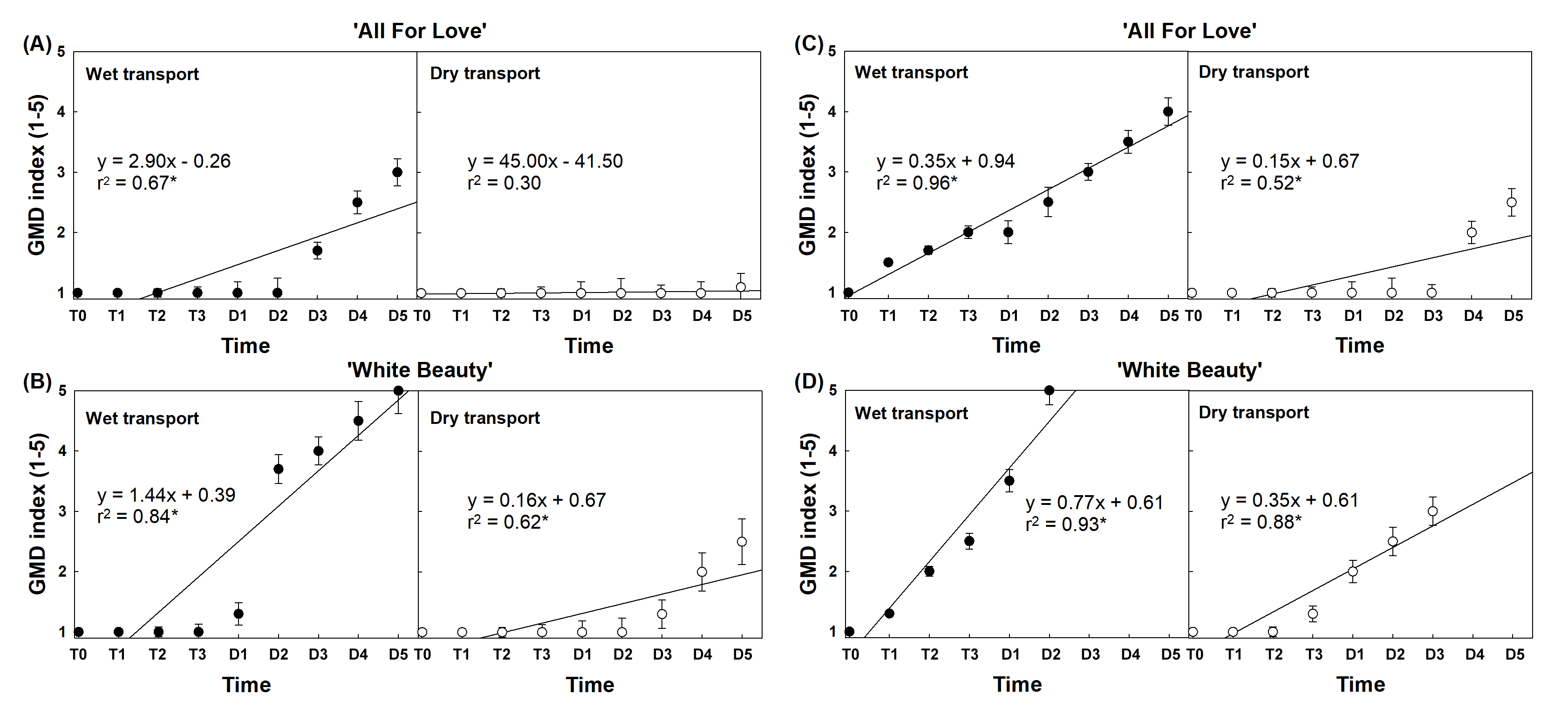
 **Supplementary Figure S2.** Simple linear regression analysis between gray mold disease (GMD) index and water stress in cut roses ‘All For Love’ (**A**) and (**C**) and ‘White Beauty’ (**B**) and (**D**). Non-inoculated (**A**) and (**B**) and inoculated (**C**) and (**D**). GMD index (1-5): 1, none; 2, slight symptoms (≤ 3 %); 3, moderate symptoms (3-10 %); 4, severe symptoms (11-50 %); and 5, death of plants (≥50 %). Cut roses were sprayed with 30 mL of *B. cinerea* conidia suspension (10^5^ conidia mL^-1^) (inoculated flowers) or 30 mL of distilled water (non-inoculated flowers). Asterisk (*) represents a significant difference at *p* = 0.05 (n = 20).

**Supplementary Figure S3.** Major factor affecting vase life of cut rose flowers (**A**) and gray mold disease (GMD) incidence in cut roses (**B**). BL, bluing; PW, petal wilting; BN, bent neck; LY, leaf yellowing; PA, petal abscission; and GMD, GMD emergence. Cut roses were sprayed with 30 mL of *B. cinerea* conidia suspension (10^5^ conidia mL^-1^) (inoculation) or 30 mL of distilled water (non-inoculation). The data were collected from 6 previous experiments in ‘All For Love’ and ‘White Beauty’ cultivars. Vertical bars indicate the SE of the mean (n = 6).


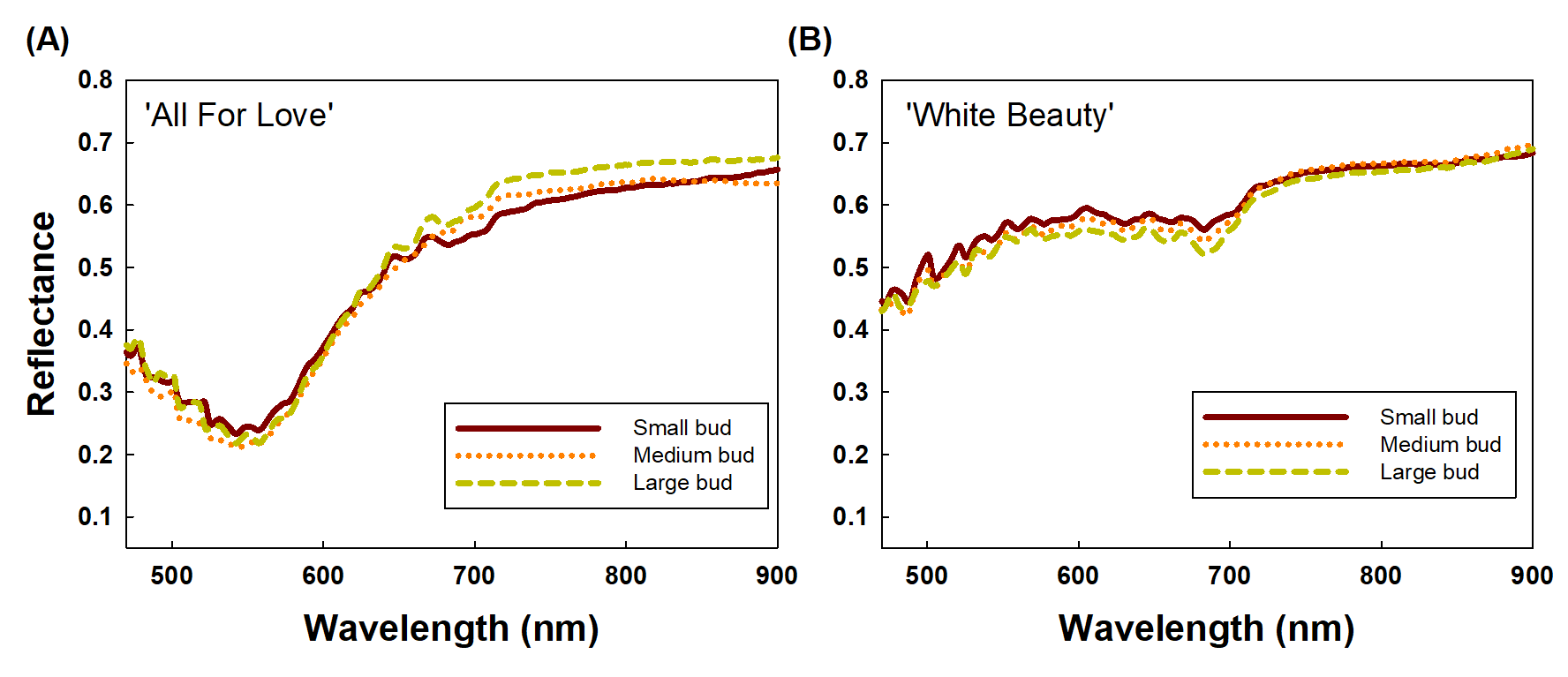
**Supplementary Figure S4.** Effects of the size of flower buds on changes in spectral curves of ‘All For Love’ (**A**) and ‘White Beauty’ (**B**) cut roses. Data were collected after wet transport treatments and on day 1 of the vase period. Data are shown as means ± SE (n = 9).


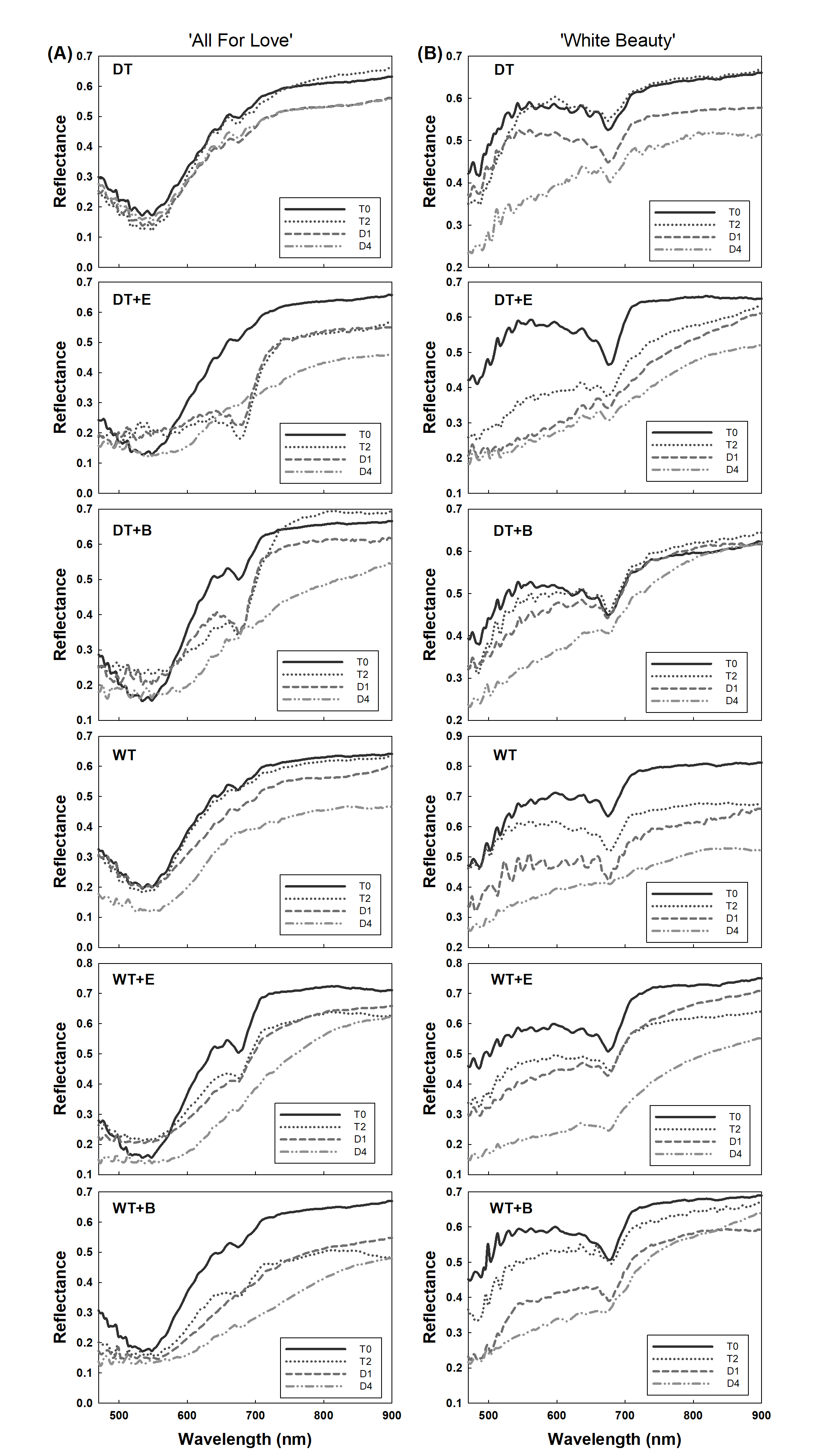


**Supplementary Figure S5.** Changes in total spectral curves of ‘All For Love’ (**A**) and ‘White Beauty’ (**B**) cut roses after transport treatments. Spectral reflectance of cut roses was collected on days 0 (T0) and 1 (T1) of the transport treatments and on days 1 (D1) and 4 (D4) of the vase period. DT, dry transport; DT+E, ethylene exposure before DT; DT+B, *B. cinerea* inoculation before DT; WT, wet transport; WT+E, ethylene exposure before WT; WT+B, *B. cinerea* inoculation before WT. Data are shown as means ± SE (n = 20).


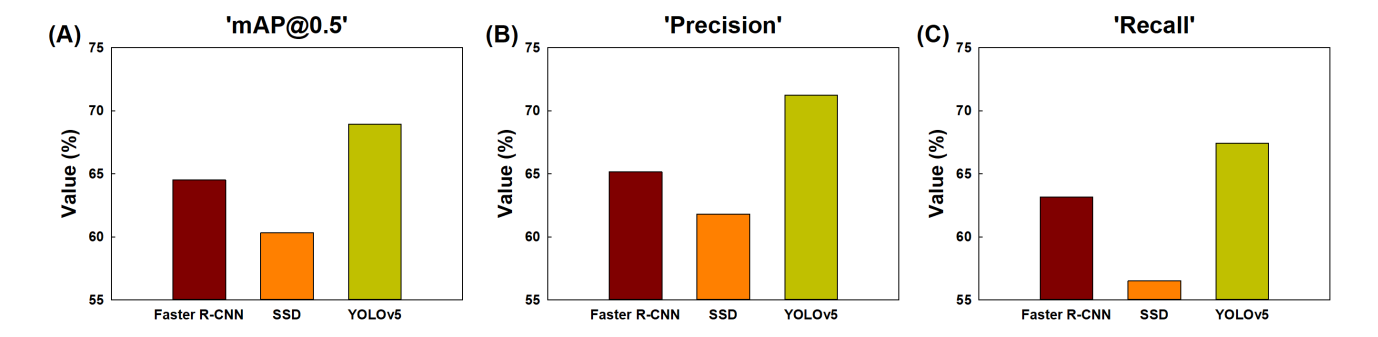


**Supplementary Figure S6.** The accuracy of the Faster R-CNN, SSD, and YOLOv5 models was evaluated by mAP (A), precision (B), and recall (C) for object detection. mAP, the evaluation index of the detection accuracy; precision, the percentage of true positives (correctly detected objects) out of all the objects that are detected; recall, the percentage of true positives (correctly detected objects) out of all the objects that exist in the dataset.


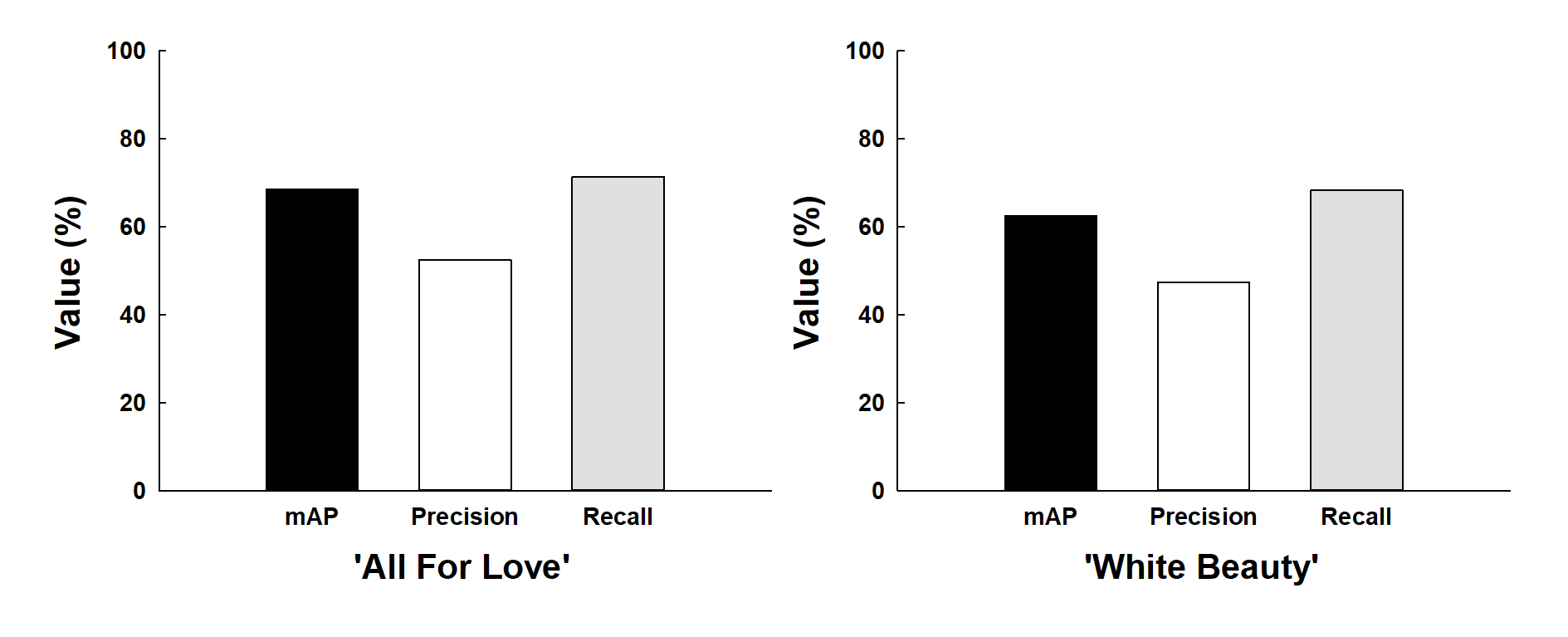


**Supplementary Figure S7.** The accuracy of the YOLOv5 model was evaluated by mAP, precision, and recall for petal wilting detection by YOLOv5. mAP, the evaluation index of the detection accuracy; precision, the percentage of true positives (correctly detected objects) out of all the objects that are detected; recall, the percentage of true positives (correctly detected objects) out of all the objects that exist in the dataset.
